# Identification of condition-specific biomarker systems in uterine cancer

**DOI:** 10.1093/g3journal/jkab392

**Published:** 2021-11-13

**Authors:** Allison R Hickman, Yuqing Hang, Rini Pauly, Frank A Feltus

**Affiliations:** 1 Department of Genetics and Biochemistry, Clemson University, Clemson, SC 29634, USA; 2 Biomedical Data Science and Informatics Program, Clemson University, Clemson, SC 29634, USA; 3 College of Science, Center for Human Genetics, Clemson University, Clemson, SC 29634, USA

**Keywords:** uterine cancer, biomarkers, gene coexpression network, gene regulatory network

## Abstract

Uterine cancer is the fourth most common cancer among women, projected to affect 66,000 US women in 2021. Uterine cancer often arises in the inner lining of the uterus, known as the endometrium, but can present as several different types of cancer, including endometrioid cancer, serous adenocarcinoma, and uterine carcinosarcoma. Previous studies have analyzed the genetic changes between normal and cancerous uterine tissue to identify specific genes of interest, including *TP53* and *PTEN*. Here we used Gaussian Mixture Models to build condition-specific gene coexpression networks for endometrial cancer, uterine carcinosarcoma, and normal uterine tissue. We then incorporated uterine regulatory edges and investigated potential coregulation relationships. These networks were further validated using differential expression analysis, functional enrichment, and a statistical analysis comparing the expression of transcription factors and their target genes across cancerous and normal uterine samples. These networks allow for a more comprehensive look into the biological networks and pathways affected in uterine cancer compared with previous singular gene analyses. We hope this study can be incorporated into existing knowledge surrounding the genetics of uterine cancer and soon become clinical biomarkers as a tool for better prognosis and treatment.

## Introduction

Uterine cancer is the most common gynecological cancer and the fourth most common cancer overall among women (Henley *et al.* 2018; Ward *et al.* 2019). The incidence of uterine cancer is increasing each year with an estimated 66,000 new cases projected for 2021 in the United States (Henley *et al.* 2018; Siegel *et al.* 2021). The 5-year survival rate for all uterine cancers is roughly 81%, but differs significantly based on cancer subtype and stage (Cantrell *et al.* 2015; Siegel *et al.* 2021).

Uterine cancer can arise in the endometrium, the lining of the uterus, or in the myometrium, the middle uterine layer composed of muscle. Endometrial cancer is more common than uterine sarcoma (cancer of the myometrium), making up roughly 85% of all uterine cancer cases (Bokhman 1983). Endometrial cancer is an overarching category of cancers, here known as UCEC. Specific subtypes include, but are not limited to, endometrioid cancer, serous adenocarcinoma, clear cell carcinoma, and uterine carcinosarcoma (UCS). Endometrioid cancer is the most common of the endometrial cancers, making up about 68% of all uterine cancer cases (Henley 2018). However, endometrioid cancer also has the best prognosis as a type 1 estrogen-dependent uterine cancer (Setiawan *et al.* 2013). In those cases the unopposed estrogen could stem from estrogen-only forms of birth control, the breast cancer drug tamoxifen, or obesity, among other causes (Sakamoto *et al.* 2002; Setiawan *et al.* 2013; Lortet-Tieulent *et al.* 2018). Serous adenocarcinoma, clear cell carcinoma, and UCS are rarer, each accounting for 3–10% of endometrial cancer cases (Cantrell *et al.* 2015; Henley *et al.* 2018). Serous adenocarcinoma and clear cell carcinoma usually arise from atrophied endometrium (Geels *et al.* 2015). These are considered estrogen-independent (type 2) and are more aggressive than type 1 uterine cancer (Setiawan *et al.* 2013). They are usually diagnosed after the cancer has spread beyond the uterus, therefore leading to a poorer prognosis (Geels *et al.* 2015). UCS is a more distinct subtype as it seems to be a cancer of both the endometrium and the myometrium (Cantrell *et al.* 2015). Based on recent research of the cellular morphology, it is more likely that the initial mutation begins in the endometrium and spreads to the myometrium (Levine 2013; Cantrell *et al.* 2015). Because of this, UCS is considered a subtype of endometrial cancer. Similar to serous adenocarcinoma and clear cell carcinoma, UCS has a poor prognosis (∼35% survival rate after 5 years) and accounts for 15% of deaths associated with uterine cancer (Vaidya *et al.* 2006; Cantrell *et al.* 2015).

To better diagnose, prognose, and treat uterine cancer, biomarkers can be used. A biomarker is a characteristic that indicates a normal or pathogenic process (FDA-NIH Biomarker Working Group 2016). Biomarkers can come in many forms, such as oxidative damage in an individual’s plasma to measure the progression of Parkinson’s disease, inflammation markers in blood to predict someone’s risk for cardiovascular disease, and characteristics of EEGs to diagnose major depressive disorder (Pearson *et al.* 2003; Bogdanov *et al.* 2008; Pillai *et al.* 2011). Many cancer-specific genetic biomarkers have also been identified. Some examples include mutations in *EGFR* and *BRAF* for nonsmall cell lung cancer, DNA methylation patterns to determine stomach cancer metastasis, and RNA expression of *SChLAP1* to predict metastasis of prostate cancer (Prensner *et al.* 2014; Wu *et al.* 2017; Pennell *et al.* 2019). For endometrial cancer, these mutations tend to arise in genes *PTEN*, *TP53*, and *CTNNB1* (Okuda *et al.* 2010). Many other genetic mutations have been identified as biomarkers of uterine cancer, some of which we rediscover here through, further ensuring the robustness of method.

The Cancer Genome Atlas (TCGA) among other projects have investigated both UCS and UCEC cancers. They have identified many singular genes that tend to be mutated across both cancer types, such as *TP53*, *PTEN*, *PIK3CA*, and *PPP2R1A* (Okuda *et al.* 2010; Levine 2013; Cherniack *et al.* 2017). While some are specific to solely UCEC (*FGFR2*, *CTNNB1*, and *POLE*), others have only been associated with UCS (*RB1*, *ZBTB7B*, and *U2AF1*) (Levine 2013; Cherniack *et al.* 2017). There have also been associations with more general characteristics. Translocations have been found with genes involved in the WNT and PI(3)K pathways, among others. Microsatellite instability is more common in endometrioid cancer compared with serous adenocarcinoma and UCS (Levine 2013). UCS and type 2 UCEC cases tend to have more CNV events compared with UCEC type I cases (Levine 2013; Zhao *et al.* 2013). Endometrial cancer can also appear in conjunction with Lynch syndrome, which is usually due to mutations in genes involved with DNA mismatch repair (Gayther and Pharoah 2010). These genes include *MLH1*, *MSH2*, *MSH6*, and *PMS2*, with mutations in *MSH6* carrying the most risk for developing endometrial cancer (Baglietto *et al.* 2010; Gayther and Pharoah 2010).

In this study, we assembled groups of related biomarkers, known as a biomarker system or biosignature, that are specific to normal uterine tissue and cancerous uterine tissue. We were interested in two types of biomarker systems: gene coexpression networks (GCNs) and gene regulatory networks (GRNs). Coexpressed genes were discovered by analyzing gene expression data and finding gene pairs that have correlated expression patterns. These coexpressed gene pairs were then assembled to form a GCN. A GCN–GRN was constructed by merging the GCN with directed tissue-specific regulatory edges [transcription factor (TF) to target gene (TR)]. These networks have been used to identify biomarkers for many complex diseases including Alzheimer’s disease, hepatocellular carcinoma, breast cancer, and kidney cancer (Chekouo *et al.* 2015; Hsu *et al.* 2019; Li *et al.* 2019; Soleimani Zakeri *et al.* 2020). Networks like these, or even subnetworks, are reproducibly better at classifying disease compared with singular genes (Chuang *et al.* 2007; Jin *et al.* 2008). Especially in complex disease, such as cancer, there are multigene pathways responsible for the diagnosis, rather than a small group of genes of interest (Zeng *et al.* 2013; Liu *et al.* 2014). The associations of those molecular interactions within each condition-specific network allows for a more comprehensive view of the cellular environment compared with singular gene associations.

Several other studies have built GCNs specific to endometrial cancer to find genes of interest. One identified several hub genes related to stage, grade, and type of endometrial cancer (Chou *et al.* 2014). Another study was interested specifically in the coexpression patterns of gene *AKT*. They were able to identify six coexpressed genes (*PBK*, *BIRC5*, *AURKA*, *GTSE1*, *KNSTRN*, and *PSMB10*), some of which were also able to predict prognosis (Huo *et al.* 2019). Other studies have discovered additional hub genes with prognostic power, *TICRR*, *PPIF*, and *ANO1* (Wang *et al.* 2019; Yang *et al.* 2021). These previous endometrial cancer studies have all used weighted GCN analysis (WGCNA) to build the GCNs, while we have taken a different approach using Gaussian Mixture Models (GMMs) prior to pairwise gene correlation tests. Our approach allows for analysis of genes that are involved in more than one biological process, and therefore have multiple expression patterns (Ficklin *et al.* 2017). In this analysis, the GMMs can differentiate between the two expression levels and test each for coexpression edges, whereas WGCNA cannot.

Uterine cancer is one of the most common cancers among women, and the incidence is increasing each year. In this study, we aimed to explore the genetic differences between normal and cancerous uterine tissue. By building uterine condition-specific biomarker systems through the use of GMMs, we can contribute to the growing literature of biological associations and changes in genetic relationships to better understand, and potentially better diagnose and treat, uterine cancer.

## Materials and methods

**Figure 1:**
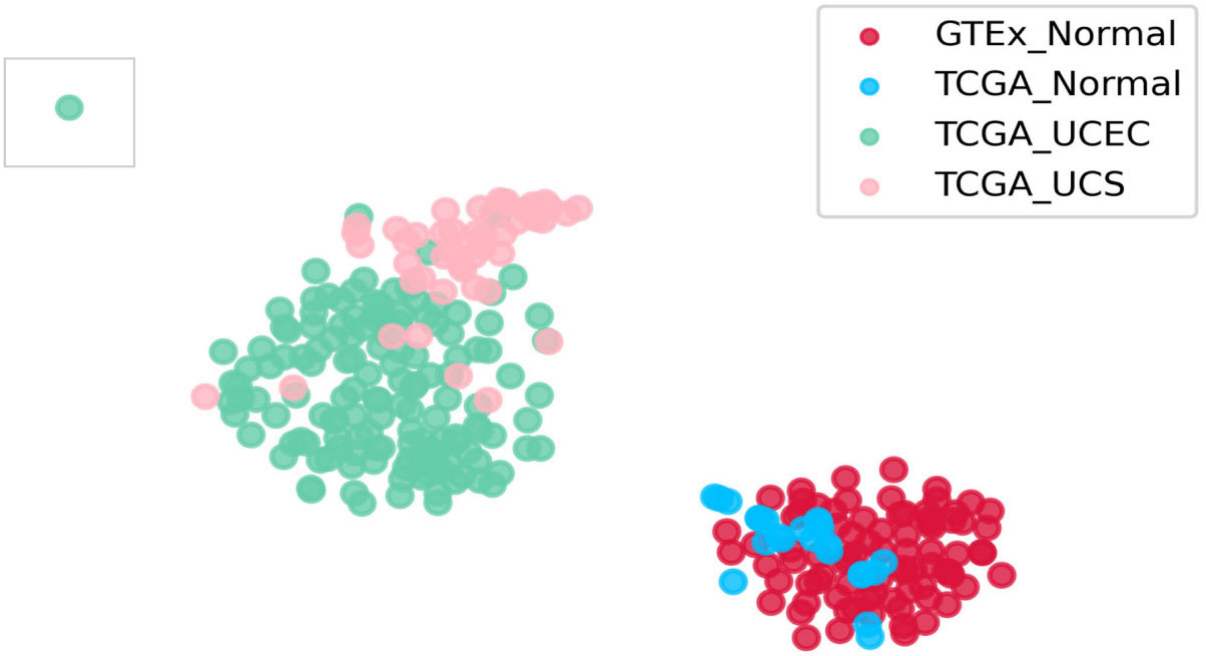
*t*-SNE plot of uterus GEM. A *t*-SNE plot of all samples included in the uterus GEM. The color of each dot represents the group that sample belongs to. The boxed outlier UCEC sample was moved closer to the other samples to avoid whitespace.

**Figure 2:**
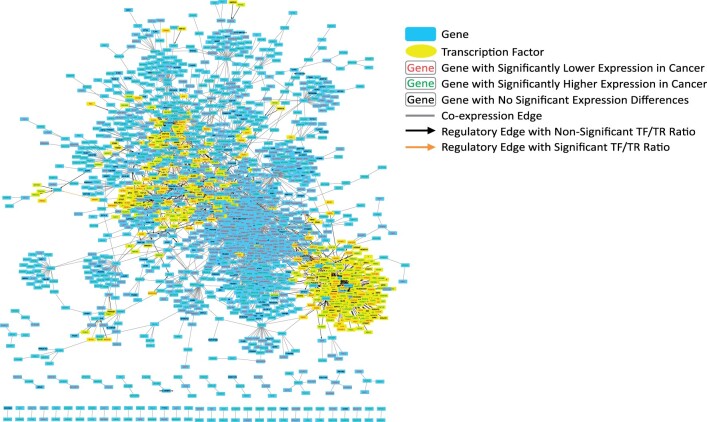
Cytoscape visualization of UCEC gene regulatory network. UCEC-specific gene regulatory network containing coexpression and regulatory edges. An interactive network is available in the Supplementary Data Cytoscape file.

**Figure 3:**
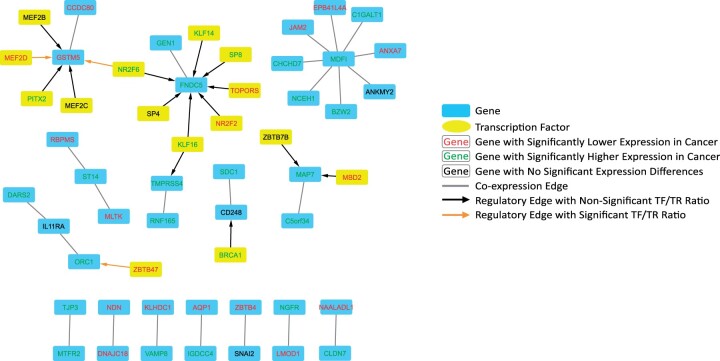
Cytoscape visualization of UCS network. UCS-specific network containing coexpression and regulatory edges. An interactive network is available in the Supplementary Data Cytoscape file.

To construct the GEM the FPKM files for all uterine samples (TCGA-Normal, TCGA-UCEC, TCGA-UCS, and GTEx-Normal) and their corresponding sample annotation matrices were downloaded and processed (https://doi.org/10.6084/m9.figshare.5330593, last accessed November 16, 2021) (Wang *et al.* 2018). The final GEM includes gene expression information across 19,304 genes in 82 GTEx Normal samples, 23 TCGA Normal samples, 141 TCGA UCEC samples, and 47 TCGA UCS samples (Figure  1). Condition-specific GCNs were then constructed using GMMs via Knowledge Independent Network Construction (KINC) software (https://github.com/SystemsGenetics/KINC, last accessed November 16, 2021). We restricted network edges to those with a *P*-value of <0.001 and consequently created condition-specific subnetworks. Differential gene expression analysis was performed between GEMs of the normal uterine tissue of GTEx (v.6) and TCGA-UCEC, as well as GTEx-Normal and TCGA-UCS. It was completed by DESeq2_1.30.1 in R 4.0 (https://bioconductor.org/packages/release/bioc/html/DESeq2.html, last accessed November 16, 2021). The RSEM counts for the analysis were obtained from https://www.nature.com/articles/sdata201861: https://doi.org/10.6084/m9.figshare.5330539, last accessed November 16, 2021. All GCN nodes, edges and their corresponding DEG status can be found in Supplementary Table S1. The results for the UCEC and UCS DESeq analyses can be found in Supplementary Tables S2 and S3, respectively. The condition-specific GCNs were then overlapped with the results from the differential expression analysis. The global attributes of each GCN network can be found in Table  1. Cytoscape was used to visualize the UCEC, UCS, and GTEx GCN networks, which can be found in Figures  2 and 3, and Supplementary Figure S1, respectively.

**Table 1:** Uterus condition-specific gene coexpression network global attributes

Sample condition	Samples	Genes	Edges	K	DEG genes	DEG edges	DEG k
GTEx_Normal	82	4372	14044	6.42	3758	12083	6.43
TCGA_UCEC	141	1496	2861	3.82	1333	2638	3.96
TCGA_UCS	47	39	24	1.23	35	19	1.09

The Glass Lab at Harvard University published tissue-associated regulatory relationships for normal tissues based on data from the GTEx project (https://sites.google.com/a/channing.harvard.edu/kimberlyglass/tools/gtex-networks, last accessed November 16, 2021) (Sonawane *et al.* 2017). From this list of regulatory edges, those enriched in uterus were extracted (*N* = 60,915). The published uterine regulatory edges were merged with each csGCN to create respective csGRNs, containing both gene coexpression edges and regulatory edges. GRN edges for the cancer networks can be found in Supplementary Table S4 and those for the GTEx GRN can be found in Supplementary Table S5. A summary of which can be found in Table  2. We were also interested in investigating possible coregulation. To do this, “triangle” relationships were extracted from the UCEC GRN. We define a triangle relationship as one in which one TF regulates two genes connected by an edge. To further investigate the regulatory edges and their differences between cancer and normal tissue, we performed a *t*-test on the FPKM ratio for the regulatory edges. For each regulatory edge the FPKM values for the TF and TR were collected for the corresponding cancer samples and GTEx-Normal samples and underwent a *t*-test as ratios (TF/TR) to see if they were significantly different (*P*-value < 1E−5) between the cancerous and normal tissues. Like the GCN analysis, the nodes of each regulatory edge were subject to a differential expression analysis, performed like above. Functional enrichment was performed on the DEGs for each csGCN (UCEC, UCS, UCEC triangle, and GTEx) using ToppFun (https://toppgene.cchmc.org/, last accessed November 16, 2021) (Supplementary Table S6). Further details on these methods can be found at http://dx.doi.org/10.17504/protocols.io.by85pzy6 (last accessed November 16, 2021).

**Table 2:** Uterus condition-specific gene coexpression network-gene regulatory network global attributes

Sample condition	Samples	Edges	Genes	TFs	TF/Gene	k	Significant Edges	DE Genes	DE TFs	DE TFs/Genes	DEG k
GTEx_Normal	82	17,547	4,281	459	91	7.26	11,193	3,475	334	83	5.75
TCGA_UCEC	141	3,976	1,462	361	34	4.28	2,994	1,302	277	31	3.72
TCGA_UCS	47	41	39	15	0	1.52	22	35	11	0	0.96
TCGA_UCEC_Triangle	141	22	7	4	0	4.00	20	7	4	0	3.64

## Results

### Segregation of cancer and control individuals based on gene expression profiles

A uterine gene expression matrix (GEM) was constructed from normalized and batch-effect-corrected FPKM files for TCGA and GTEx uterine samples, published by Wang *et al.* (2018) (https://doi.org/10.6084/m9.figshare.5330593, last accessed November 16, 2021). The GEM includes gene expression information for 19,304 genes across 82 GTEx Normal samples, 23 TCGA Normal samples, 141 TCGA-UCEC samples, and 47 TCGA-UCS samples. To visualize the GEM, we used *t*-distributed stochastic neighbor embedding (*t*-SNE), a technique used to visualize high-dimensional data in a 2D scatterplot (Van der Maaten and Hinton 2008). The *t*-SNE visualization of the GEM can be found in Figure  1, which shows the spatial representation of the 293 samples, based on their gene expression profiles. The cancer samples, TCGA-UCEC and TCGA-UCS (represented by green and pink dots, respectively), can be seen segregating separate from the normal samples, GTEx-Normal and TCGA-Normal (red and blue, respectively). One TCGA-UCEC outlier was boxed and brought in closer to allow for better visibility of the two main clusters.

### Constructing condition-specific GCNs

To construct condition-specific GCNs (csGCNs) from the uterine GEM, we first used Knowledge Independent Network Construction (KINC) (https://github.com/SystemsGenetics/KINC, last accessed November 16, 2021). KINC identifies gene coexpression edges that are condition-specific. The conditions of interest for this study are normal uterine tissue (GTEx-Normal), and two types of cancer (TCGA-UCEC and TCGA-UCS). KINC uses GMMs to cluster samples, then performs a pairwise gene correlation analysis on each cluster. Only clusters with greater than 25 samples were considered, so the TCGA-Normal samples were not used for any further analyses. Condition-specific gene pairs with a Spearman correlation <−0.5 or >0.5 were considered for further testing. Low-powered edges within each cluster were removed, and a hypergeometric test was used to assign *P*-values to those remaining. Condition-specific networks were created and edges within each network with a *P*-value >0.001 were removed. Biased edges were also removed, and remaining edges were ranked based on their *P*-values and similarity scores. Through this process, KINC built three csGCNs: GTEx-Normal (14,044 edges and 4,372 genes), TCGA-UCEC (2,861 edges and 1,496 genes), and TCGA-UCS (24 edges and 39 genes) (Table  1). To get a better idea of network connectivity, we multiplied the number of edges by two and divided that number by the number of nodes to find *k*. The GTEx-Normal had the highest average connectivity (*k *=* *6.42), followed by UCEC (*k *=* *3.82), and finally UCS (*k *=* *1.23) (Table  1). Further details on GCN construction are provided at http://dx.doi.org/10.17504/protocols.io.by85pzy6 (last accessed November 16, 2021).

Before further investigating these csGCNs, we wanted to analyze the gene expression patterns of the genes within the networks. More specifically, we were interested in determining if the genes within the csGCNs were differentially expressed between each respective cancer condition and GTEx-Normal. A total of 20,242 genes were analyzed for both UCEC and UCS. The differential gene expression analysis was performed using DESeq2_1.30.1 in R 4.0 (https://bioconductor.org/packages/release/bioc/html/DESeq2.html, last updated November 16, 2021). Genes had to have expression values for at least 50 samples for each analysis. In total, 14,551 and 13,898 genes were found to be significantly differentially expressed in the comparison of GTEx-Normal with UCEC and UCS, respectively (adjusted *P*-value <0.001).

The genes within each GCN were merged with the differential expression analysis to denote the differential expression status of each csGCN gene. Of the 1,496 genes in the UCEC-specific network, 1,333 were differentially expressed, accounting for roughly 89% of the genes. In the UCS-specific network 35 of the 39 genes were differentially expressed, accounting for 90% of the genes. For the UCEC csGCNs, when observing only the DEGs and DEG edges (edges in which both nodes are DEGs), there is a slight increase in *k*, of which can also be observed in GTEx-Normal. When similarly assessing UCS, *k* slightly decreases. The csGCNs are represented in Figure  2 (UCEC), Figure  3 (UCS), and Supplementary Figure S1 (GTEx-Normal). In the network visualizations, the genes are represented as blue nodes, and coexpression edges are depicted as gray lines. Furthermore, the text color of each node denotes if the gene is upregulated in cancer (green), downregulated in cancer (red), or was not significantly different between cancer and GTEx-Normal (black). The attributes of each GCN network can be found in Table  1. The complete list of condition-specific edges and each node’s DEG status can be found in Supplementary Table S1.

Directed regulatory edges derived from the GTEx database and published by the Glass Lab at Harvard were downloaded (https://sites.google.com/a/channing.harvard.edu/kimberlyglass/tools/gtex-networks, last updated November 16, 2021). We extracted the uterine regulatory edges and merged them with the coexpression edges in our csGCNs to create condition-specific GRNs (csGRNs). Similar to the GCN construction, the TFs were also subject to the differential expression analysis. Figures  2 and 3 both contain these regulatory edges (denoted as arrowed edges) for UCEC and UCS, respectively. Their regulation pattern in cancer is denoted like that of the genes described above (red: significantly downregulated in cancer; green: significantly upregulated in cancer; black: no significant difference in expression). A summary of the GCN–GRNs can be found in Table  2.

### Integration of regulatory edges to construct condition-specific GRNs

Another step we included in GRN assembly was a ratio analysis comparing the expression ratio of the TF to the TR. For each regulatory edge, the TF/TR ratio was compared between GTEx-Normal samples and the respective cancer samples using a Student’s *t*-test. In Figures  2 and 3, the regulatory edges with significantly different ratios (*P*-value <1E−5) are shown as orange arrowed edges, and those without a significantly different ratio between normal and cancer conditions are shown as black arrowed edges. More detailed descriptions of these analyses can be found at http://dx.doi.org/10.17504/protocols.io.by85pzy6 (last updated November 16, 2021).

**Figure 4:**
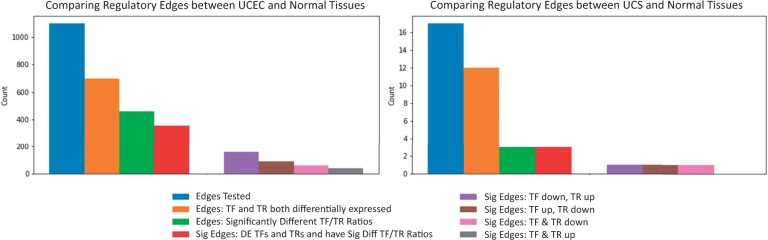
Expression patterns of transcription factors and target genes in cancer-specific regulatory edges. Distribution of expression patterns and ratio analyses results for regulatory edges in UCEC and UCS networks compared with GTEx.

Of the 1,101 regulatory edges investigated for the UCEC GRN, 356 had a significantly different TF/TR ratio between GTEx-Normal and UCEC samples and consisted of a TF and TR that were both differentially expressed. Because those edges meet three separate requirements (differentially expressed TF, differentially expressed TR, and significant TF/TR ratio), they will be referred to as “significant” regulatory edges. A summary of the distribution of edges across the four possible expression patterns (TF and TR up, TF and TR down, TF up and TR down, TF down and TR up) can be found in Figure  4. Of the 356 significant edges, 163 had a lower TF expression and higher TR expression in UCEC. The least common TF and TR expression patterns found in the UCEC network was one in which both the TF and TR were both upregulated. In the UCS-specific GRN there were only three regulatory edges that met the significance criteria, none of which had the same TF and TR expression pattern (one TF down and TR up edge, one TF up and TR down edge, one TF and TR down edge). A heatmap visualization with DEG status of TFs and TRs, TF and TR expression pattern in cancer, and designation of significant TF/TR ratios can be seen in Supplementary Figure S2.

**Figure 5:**
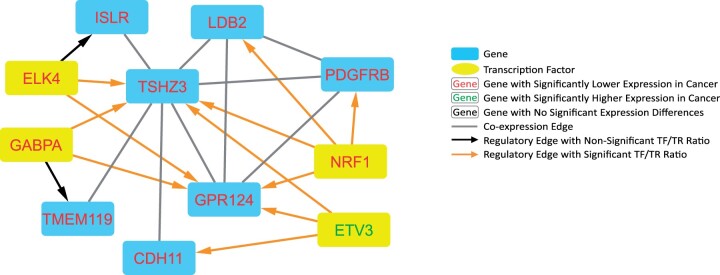
Cytoscape visualization of UCEC triangle network. A UCEC-specific triangle network in which transcription factors are associated with both nodes of a coexpression edge.

Within these csGRNs, we were particularly interested in potential co-regulation relationships, so we selected “triangle” subnetworks for further study. Here, we define a triangle relationship as one in which a TF regulates two TRs that are connected by an csGCN edge. These triangle relationships were extracted from the UCEC GRN to create what we have deemed the “UCEC triangle GRN.” As seen in Figure  5, the UCEC triangle GRN is made up of four TFs and seven genes, almost all of which have a significantly lower expression in UCEC compared with normal, as noted by the red gene names. In this triangle network there is one highly connected core group including genes *TSHZ3*, *GPR124*, *LDB2*, and *PDGFRB*, and TF *NRF1*. All the gene pairs within that core group are co-expressed, and NRF1 has a significant TF/TR ratio with each gene. Likely because of its smaller size, no triangle relationships were present in the UCS GRN, so therefore no corresponding triangle GRN was assembled. The attributes of the UCEC triangle GRN can be found in Table  2 and the DNA mutation rates for these genes in UCEC can be found in Supplementary Table S7.

### Functional enrichment of differentially expressed genes within condition-specific GCNs

Functional enrichment was performed for the genes within the UCEC GCN, the UCS GCN, and the UCEC triangle GRN using ToppFun (https://toppgene.cchmc.org/enrichment.jsp). ToppFun uses a set of genes as input to identify associations of those genes with published annotations, such as microRNAs, phenotypes, and cell types. The DEG list for each csGCN was used as the input, and the default full gene set as the background. The UCEC DEG list (*n* = 1,333 genes) had 16,305 significant associations (*q*-val FDR B&H < 1E−5). Several associations of interest included ovarian cancer (*q*-val FDR B&H = 8.51E−73), endometrial cancer (*q*-val FDR B&H = 2.39E−54), cervical cancer (*q*-val FDR B&H = 3.03E−45), and normal endometrial tissue (*q*-val FDR B&H = 6.56E−43). Functional enrichment of the UCS DEG list (n = 35) yielded four significant associations (*q*-val FDR B&H < 1E−5), of which included basal lung cells (*q*-val FDR B&H = 3.46E−6), immune cells (*q*-val FDR B&H = 3.46E−6), and “genes upregulated in uterus upon knockout of *BMP2*” (*q*-val FDR B&H = 6.62E−6). Functional enrichment of the genes within the UCEC triangle GRN was also performed, which yielded 110 significant associations (*q*-val FDR B&H < 1E−5). These associations included many TCGA cancers, like that of the bladder, stomach, lung, and ovary, as well as mesenchymal cells. The functional enrichment associations for the DEGs within the UCEC, UCS, UCEC triangle, and GTEx networks can be found in Supplementary Table S6.

## Discussion

In this study, we constructed uterine condition-specific gene relationship networks where genes are defined by coexpression and regulatory edges. Our csGCN search space was a unified uterine GEM containing cancerous (TCGA-UCEC and TCGA-UCS) and normal (TCGA-Normal and GTEx-Normal) tissue samples. The directed GRN graph was derived from annotated uterine gene regulatory relationships using GTEx-Normal tissue samples. Thus, our final gene relationship graph for uterine derived normal and tumor samples is built from the appropriate tissue.

As shown in Figure  1, the global RNA expression profiles for normal and diseased uterine samples segregated due to the overall gene expression patterns, with the exception of one TCGA-UCEC outlier that can be seen in the top left corner of the *t*-SNE visualization. To identify genetic expression subsystems that discriminate between conditions (*i.e.*, biomarker systems), we assembled csGCNs for two uterine cancers (UCEC and UCS) and normal uterine tissue (GTEx-Normal). The coexpression edges were discovered using KINC, a program that finds gene pairs based on correlations in their expression patterns within each respective condition. TCGA-Normal samples could not be processed through KINC due to the limited number of samples, so therefore, they were excluded from the remaining analyses. To further validate these csGCNs, a differential expression analysis (DESeq2) was used to find coexpression edges that have a significantly different expression pattern between cancer and normal samples. The DESeq2 results can be found in Supplementary Tables S2 and S3, a summary of which can be found in Table  1.

We then incorporated directed regulatory edges (TF to TR) into each respective GCN to build csGRNs. This was done by adding the regulatory edges into each GCN if the TR was present in the network. In the UCEC GRN, there were several instances where one TF was associated with both nodes of a coexpression edge, a relationship we dubbed a “triangle.” These potential coregulation triangle relationships were extracted, which constitutes another GRN, the UCEC triangle GRN. Finally, we wanted to further investigate these regulatory edges by comparing the TF/TR FPKM ratio for the cancer and normal samples, then use a *t*-test to determine if the ratios between the two groups were significantly different. This was completed for the regulatory edges in the UCEC, UCS, GTEx, and UCEC triangle GRNs. For the GTEx GRN, the genes and TFs had to be differentially expressed in both UCS and UCEC, and regulatory edges had to have a significantly different TF/TR ratio compared with UCEC and UCS. The regulatory edges with significantly different TF/TR ratios can be seen as orange arrowed edges in Figure  2 (UCEC GRN), Figure  3 (UCS GRN), Figure  5 (UCEC triangle GRN), Supplementary Figure S1 (GTEx GRN) and are documented in Supplementary Table S4 (Cancer GRN edges) and Supplementary Table S5 (GTEx GRN edges).

### UCEC biomarker systems

The UCEC triangle GRN was constructed due to our interest in possible coregulation of csGCN edges. This triangle subnetwork drastically reduced the size of the overall UCEC GRN, allowing us to further investigate these genes of interest. All the genes and TFs in the UCEC triangle GRN are differentially expressed, and all, except *ETV3*, had a significantly lower expression in UCEC. Five of the seven genes in the UCEC Triangle GRN are not included in the GTEx-Normal GCN, indicating their expression patterns change in UCEC. *TMEM119* and *CDH11* are the exceptions, each with two coexpression edges in the normal uterine network. Meanwhile all seven genes are highly connected in the UCEC-specific GCN, associated with a total of 106 other genes (mean = 28 edges/gene, median = 20 edges/gene). *GPR124* and *CDH11* have the highest and lowest connectivity with 66 and 6 coexpression edges, respectively. In the UCEC triangle GRN there are also four TFs, each with varying connectivity across the UCEC and GTEx networks. *ETV3* and *NRF1* have the highest connectivity, each regulating 13 genes with significant TF/TR ratios. The gene with the lowest number of significant regulatory associations in UCEC was *GABPA* with seven edges. In the GTEx GRN *NRF1* has the most significant associations at 13 genes, while *ELK4* has the fewest with four significant associations. It is possible that these changes in coexpression patterns between the UCEC and GTEx-Normal GCNs are due to mutations in the *cis* or *trans* DNA sites.

In the UCEC triangle GRN there is a highly connected core group composed of TF *NRF1* and genes *GPR124*, *LDB2*, *TSHZ3*, and *PDGFRB*. *NRF1* works in cell homeostasis through transcriptional control mechanisms and has previously been implicated in multiple types of cancer (Bhawe and Roy 2018; Yuan *et al*. 2018). *GPR124*, a G-protein coupled receptor, is involved in the WNT pathway (Zhou and Nathans 2014). Mutations of other genes in this pathway have been associated with endometrial cancer (Levine 2013). *TSHZ3* has been shown to be downregulated in breast, prostate, and colorectal cancers, similar to our results (Yamamoto *et al.* 2011; Zhou *et al.* 2021). *LDB2* also works in transcriptional regulation and has been found to have a lower expression in liver cancer as well (Yu *et al.* 2017). *PDGRFB* was recently associated with *TMEM119*, another member of the UCEC triangle GRN, and *AKT*, a previously identified endometrial cancer hub gene, in ovarian cancer (Huo *et al.* 2019; Sun *et al.* 2021).

KINC, DESeq2, and the TF/TR ratio analysis have all been completed based on RNA expression values found in the uterine GEM. The Genomic Data Commons Data Portal (GDC) contains information on DNA mutation rate and CNV occurrence rate for the UCEC samples from our analysis, as well as 389 additional samples that have been added since the uterine GEM was created by the Wang Lab. This platform allows for comparison of mutation rates for our genes of interest with other cancer projects, including other TCGA projects. The number of projects that investigated these genes ranges from 21 to 35. Of the 11 nodes (7 genes, 4 TFs) in the UCEC Triangle GRN, nine had the highest mutation rates in TCGA-UCEC. For *LDB2* and *TMEM119* the highest mutation rate was in TCGA-SKCM, a melanoma study and was followed immediately by TCGA-UCEC. Both genes have been implicated in other cancers, and therefore are not solely specific to endometrial cancer (Yu *et al*. 2017; Zheng *et al*. 2018). A summary of the DNA mutations rates of these genes can be found in Supplementary Table S7.

When investigating the CNV occurrence rate for the UCEC Triangle GRN genes and TFs, UCEC consistently had a lower rate of CNV events at those genes compared with UCS. For example, when investigating *TSHZ3* TCGA-UCEC ranked seventh highest in CNV occurrence rate with 60 events out of 510 cases (11.76%), while TCGA-UCS ranked no. 1 with 21 events out of 56 cases (37.5%). While initially we expected UCEC CNV events to be more common, given they are specific to a UCEC network, this seems to reflect the results in TCGA’s comprehensive UCEC study where UCS samples were significantly more likely to be characterized by CNV events compared with endometrioid samples (Levine 2013). These CNV rates can also be found in Supplementary Table S7.

There are several previously reported genes with DNA mutations specific to UCEC including *FGFR2*, *CTNNB1*, and *POLE* (Levine 2013). *FGFR2* and *CTNNB1* were not included in the final DEG results and had no condition-specific edges. *POLE* was not considered a DEG in the UCEC *vs* GTEx-Normal analysis (*P*-adj = 0.033), but did have coexpression edges with *PHF1*, *ABRACL*, *SPAG5*, *STC2*, and *CMYA5* in the GTEx-Normal GCN. *POLE* was not present in the UCEC-specific GCN.

### UCS biomarker systems

The TCGA landmark paper also identified several genes specific to UCS with common mutations (Cherniack *et al.* 2017). These include *RB1*, *ZBTB7B*, and *U2AF1*, each of which was found to be mutated in 4−11% of samples. In our analysis *RB1* was found to be differentially expressed in UCS samples (*P*-adj = 4.32E−5) but did not have any UCS-specific edges. Meanwhile, *U2AF1* was not found to be differentially expressed in UCS (*P*-adj = 6.36E−3). Like the other genes listed above, it is possible that the DNA mutations described in previous literature did not result in a significant difference in the mRNA expression. *ZBTB7B* was not included in the final differential expression results, due to pre- or postanalysis filtering.

### Combined UCEC and UCS biomarker systems

Previous studies have found several commonly mutated genes for both UCEC and UCS tumors: *TP53*, *PTEN*, *PIK3CA*, *PPP2R1A*, *FBXW7*, and *KRAS* (Cherniack *et al.* 2017). Of these six genes, five of them (*TP53*, *PTEN*, *PPP2R1A*, *FBXW7*, and *KRAS*) were all found to be differentially expressed in our study. *PIK3CA* was not included in the final differential expression results. However, when looking between condition-specific networks we only observe a change in gene coexpression patterns for the *KRAS* gene when comparing UCEC and GTEx samples. In UCEC samples *KRAS* is co-expressed with *CDON* and *FZD4*, while it is co-expressed with *TSPAN2* in the GTEx-Normal samples. One possible mechanism that could be attributed to this change is through the association of TFs. However, there were no known TFs that regulate *KRAS* in the uterus. Instead, the change in the co-expression relationships could be due to the mutation rate, as *KRAS* is mutated in roughly 25% of UCEC cases (Grossman *et al.* 2016). Other possibilities include epigenetic modifications, associated microRNAs, or other cellular interactions, which could be investigated in a future study. Regarding the remaining genes listed above, there were no condition-specific edges. While they were differentially expressed between UCS and GTEx-Normal, KINC did not find any changes in gene co-expression relationships between UCS and normal uterine tissue. It is possible that although those genes are relatively more frequently mutated in uterine cancer, there are no changes in the gene expression patterns.

Other genes have also been previously identified that had mutations in both UCEC and UCS (Cherniack *et al.* 2017). These include *CDH4*, *ARID1A*, *ARHGAP35*, *SPOP*, and *PIK3R1*. *CDH4* was not found to be differentially expressed in UCEC or UCS samples. *ARHGAP35* was not found to be differentially expressed in UCS samples and was dropped in pre- or postprocessing for the UCEC differential expression analysis. *ARID1A* was also absent from the differential analysis results for both UCEC and UCS analyses. *SPOP* was found to be differentially expressed in both UCS and UCEC samples and had several condition-specific relationships. In UCEC *SPOP* is coexpressed with *TOM1L1* and *C2ORF15*, and coexpressed with many genes in GTEx-Normal (*PPP1R12B*, *H2AFY*, *BZW2*, *TPM3*, *LRRC59*, *COPG1*, *HLF*, and *EIF4G1*). *SPOP* has no associations of annotated TFs in the uterus, so it is possible that the changes in coexpression edges could be due to the mutations. *PIK3R1* has been shown to have mutations in both UCEC and UCS, but more commonly in UCEC (Cherniack *et al.* 2017). In our study, *PIK3R1* was differentially expressed in both UCEC (*P*-adj = 2.41E−4) and UCS (*P*-adj = 3.08E−12) but did not have any condition-specific coexpression edges.

Uterine cancer is one of the leading cancers among women in the United States. Because of this, we were interested in using bioinformatic tools to investigate the genes and their relationships that differentiate endometrial cancer and UCS from normal uterine tissue. We accomplished this by constructing and investigating GCNs and GRNs for normal and cancerous conditions in the uterus, and validating them using differential expression, functional enrichment, and a ratio analysis of expression data for regulatory relationships. These networks add to the growing knowledge of uterine cancer biomarker systems and help elucidate the altered biological pathways that occur. In addition, we aim to better characterize uterine cancer by pursuing further investigations into the two distinct types of endometrial cancer, specifically endometrioid carcinoma and serous adenocarcinoma. In total we hope this knowledge can be used to better prognose and develop treatments for individuals impacted by these uterine cancers in the future.

## Data availability

The Supplementary tables containing full networks are available via figshare: https://doi.org/10.25387/g3.16869645.
